# Peroral Clove Essential Oil Treatment Ameliorates Acute Campylobacteriosis—Results from a Preclinical Murine Intervention Study

**DOI:** 10.3390/microorganisms9040735

**Published:** 2021-03-31

**Authors:** Stefan Bereswill, Soraya Mousavi, Dennis Weschka, Agnes Buczkowski, Sebastian Schmidt, Markus M. Heimesaat

**Affiliations:** 1 Gastrointestinal Microbiology Research Group, Institute of Microbiology, Infectious Diseases and Immunology, Charité—Universitätsmedizin Berlin, Corporate Member of Freie Universität Berlin, Humboldt-Universität zu Berlin, and Berlin Institute of Health, 12203 Berlin, Germany; stefan.bereswill@charite.de (S.B.); soraya.mousavi@charite.de (S.M.); dennis.weschka@charite.de (D.W.); buczkowski@hofmannundsommer.de (A.B.); s.schmidt@hofmannundsommer.de (S.S.); 2 Hofmann & Sommer GmbH und Co. KG, Büro Berlin, 12489 Berlin, Germany

**Keywords:** clove essential oil, enteropathogenic infection, *Campylobacter jejuni*, immune-modulatory effects, microbiota-depleted IL-10^−/−^ mice, acute campylobacteriosis model, host-pathogen interaction, preclinical intervention study, natural antibiotics-independent compounds, eugenol

## Abstract

*Campylobacter* (*C.*) *jejuni* infections pose progressively emerging threats to human health worldwide. Given the rise in antibiotic resistance, antibiotics-independent options are required to fight campylobacteriosis. Since the health-beneficial effects of clove have been known for long, we here analyzed the antimicrobial and immune-modulatory effects of clove essential oil (EO) during acute experimental campylobacteriosis. Therefore, microbiota-depleted interleukin-10 deficient (IL-10^−/−^) mice were perorally infected with *C. jejuni* and treated with clove EO via drinking water starting on day 2 post-infection. On day 6 post-infection, lower small- and large-intestinal pathogen loads could be assessed in clove EO as compared to placebo treated mice. Although placebo mice suffered from severe campylobacteriosis as indicated by wasting and bloody diarrhea, clove EO treatment resulted in a better clinical outcome and in less severe colonic histopathological and apoptotic cell responses in *C. jejuni* infected mice. Furthermore, lower colonic numbers of macrophages, monocytes, and T lymphocytes were detected in mice from the verum versus the placebo cohort that were accompanied by lower intestinal, extra-intestinal, and even systemic proinflammatory cytokine concentrations. In conclusion, our preclinical intervention study provides first evidence that the natural compound clove EO constitutes a promising antibiotics-independent treatment option of acute campylobacteriosis in humans.

## 1. Introduction

The Gram-negative thermophilic and microaerophilic bacterial species of the genus *Campylobacter* are causative agents of foodborne enteritis [1]. The incidences of human campylobacteriosis particularly due to *Campylobacter* (*C.*) *jejuni* infections rise all around the globe and are responsible for socioeconomic burdens in the range of billions of dollars annually [2,3]. Since poultry meat products represent major sources of human infections a worldwide “One World—One Health” approach is required to reduce campylobacteriosis cases [1,4]. The most prevalent foodborne infection chain from chickens to humans is established upon the symptomless colonization of the avian intestinal tract by *C. jejuni*. The absence of clinical signs upon *C. jejuni* colonization is partly due to the weak responses of birds to bacterial endotoxins including lipo-oligosaccharides (LOS) derived from *C. jejuni* which represent truncated variants of lipo-polysaccharide (LPS) produced by most other Gram-negative bacteria [4,5,6]. However, the LOS responses in humans are far more pronounced as compared to birds and therefore, *C. jejuni* infected patients develop a symptom complex designated as campylobacteriosis which is clinically characterized by acute bacterial enteritis with severe diarrhea, abdominal cramping, nausea, and vomiting [4,5,7,8,9]. A recent study confirmed the central role of the pathogenic endotoxin in the initiation of campylobacteriosis given that in *C. jejuni* infected patients the diarrhea was caused by a LOS-induced proinflammatory cytokine storm resulting in intestinal apoptosis, epithelial barrier destruction and sodium malabsorption [10].

The helical shape and the extraordinary motility of *C. jejuni* bacteria are both required for the induction of inflammatory host responses mounting in acute campylobacteriosis [7,11]. Furthermore, bacterial entry to intestinal tissues requires *C. jejuni* adhesion and invasion factors, which have been investigated intensively at the molecular level [7]. The absence of a potent exotoxin produced by all *C. jejuni* isolates hinders effective vaccination and indicates that pathogenesis of campylobacteriosis is driven by endotoxins resulting in hyper-acute inflammation due to excessive reactions of host immunity. In humans, *C. jejuni* invasion activates innate immune responses resulting in production of reactive oxygen species (ROS) and pro-inflammatory mediators which further promote enteritis by apoptosis and ultimately, by intestinal tissue destruction, both of which constituting histopathological key indicators of acute campylobacteriosis [8,9,10]. The central role of innate immune activation by *C. jejuni* endotoxins in induction and progress of human campylobacteriosis is further supported by the fact that distinct molecule variants of the surface-located LOS molecules trigger post-infectious sequelae such as Guillain-Barré-syndrome or reactive arthritis [7]. The distinct modification of LOS of the infecting *C. jejuni* strain by sialylation is significantly associated with the severity of the preceding enteritis and with the occurrence of these autoimmune diseases weeks to months after the infectious event [12]. Thus, *C. jejuni* LOS triggers both the severity of enteritis and the development of post-infectious morbidities. 

Given the essential role of the host innate immunity in *C. jeuni* induced disease, pharmaceutical intervention strategies should aim at alleviating the proinflammatory responses during campylobacteriosis by immune-modulation and dampening of ROS induced apoptosis, which in turn, prevents further tissue destruction. This necessitates a convenient and reliable murine infection and inflammation model which was recently developed by the generation of microbiota-depleted mice sensitized to LOS by defects in genes for interleukin- (IL-)10 or single-Ig-interleukin-1 related receptor (SIGIRR) [13,14,15,16]. Microbiota depletion by antibiotic treatment has been shown essential to overcome the colonization resistance against *C. jejuni,* which is mediated by the murine commensal gut microbiota [17]. In particular, the *C. jejuni* infected microbiota-depleted IL-10^−/−^ mice were further standardized and are presently used as a validated model to study host-pathogen interactions during acute campylobacteriosis (reviewed by Mousavi et al., [15,16]). The intestinal immune responses and clinical signs in microbiota-depleted IL-10^−/−^ mice are very similar to symptoms observed in humans suffering from acute campylobacteriosis, which also holds true for intestinal immunohistopathological changes characterized by innate immune cell accumulation, apoptosis, and tissue destruction [9,18,19]. Given that non-motile *C. jejuni* mutants were shown to be apathogenic in microbiota-depleted IL-10^−/−^ mice despite high intestinal bacterial loads [20], and *C. jejuni* induced enteritis was ameliorated in animals lacking the LOS sensor toll-like receptor (TLR) -4, this murine model was chosen for the evaluation of novel pharmaceutical intervention strategies for prophylaxis and treatment of campylobacteriosis (reviewed by Mousavi et al., [15,16]). The effective treatment of acute *C. jejuni* induced enteritis in microbiota-depleted IL-10^−/−^ mice by rapamycin indicates that the suppression of the excessive innate immune responses constitutes a promising approach for novel intervention strategies [21]. By following this rationale, we have applied this campylobacteriosis infection model for the investigation of disease-alleviating properties of various non-toxic immune-modulating substances [16,22] including vitamin D [23], vitamin C [24], carvacrol [25], neuropeptides [26,27], urolithin-A [28] and cardamom [15] to date.

To elaborate additional options for prophylaxis and treatment of campylobacteriosis, our group is searching for pharmaceutically documented non-toxic natural products, which combine potent anti-inflammatory and antioxidant effects with antimicrobial activity against *C. jejuni* to avoid intestinal pathogen overgrowth and invasion. Furthermore, a treatment option of interest should in parallel dampen innate immune responses with a minimal risk of unwanted side effects. Clove essential oil (EO) appeared as a promising candidate since it has been shown to exert antimicrobial activity against *C. jejuni* [29] and other bacterial pathogens mainly by disruption of the outer membrane and by the production of intracellular ROS in the bacteria [30,31]. Furthermore, recent studies revealed that coincubation of *C. jejuni* with clove EO and its major compound eugenol resulted in compromised pathogenic motility, disturbed spiral-shaped morphology, and diminished virulence factor expression, all of which required for induction of campylobacteriosis [11,31,32,33,34]. Moreover, the pronounced ROS scavenging properties of eugenol, which is known for its health-beneficial including anti-inflammatory effects since centuries [35] have the potential to dampen innate immune responses in human tissues which have been proven effective in many other acute and chronic inflammatory diseases [36]. Finally, treatment studies revealed that clove EO and eugenol ameliorate chemically induced colitis in rats [37,38]. All these multifaceted beneficial effects of clove EO prompted us to evaluate this natural product as potential treatment option of campylobacteriosis in a preclinical setting for the first time by using *C. jejuni* infected microbiota-depleted IL-10^−/−^ mice. 

## 2. Materials and Methods

### 2.1. Ethical Statement

All animal experiments were carried out according to the European animal welfare guidelines (2010/63/EU) following approval by the commission for animal experiments (“Landesamt für Gesundheit und Soziales”, LaGeSo, Berlin; registration number G0104/19). Animal welfare was monitored daily by assessing the clinical conditions of each mouse. 

### 2.2. Microbiota-Depleted IL-10^−/−^ Mice

IL-10^−/−^ mice (C57BL/6j background) were bred in the Forschungsinstitute für Experimentelle Medizin, Charité—Universitätsmedizin Berlin, Germany. Under standard conditions (i.e., 22–24 °C room temperature, 55 ± 15% humidity, 12 h light/12 h dark cycle) mice were housed in cages including filter tops within an experimental semi-barrier and had free access to autoclaved water (*ad libitum*) and standard chow (food pellets: ssniff R/M-H, V1534-300, Sniff, Soest, Germany). To eradicate the commensal gut microbiota, 3-week-old female and male mice were exposed to a broad-spectrum antibiotic treatment as described earlier [39,40]. Briefly, mice were transferred to sterile cages (maximum of 3–4 animals per cage) and received an antibiotic cocktail for 8 weeks by adding ampicillin plus sulbactam (1 g/L; Dr Friedrich Eberth Arzneimittel, Ursensollen, Germany), vancomycin (500 mg/L; Hikma Pharmaceuticals, London, UK), ciprofloxacin (200 mg/L; Fresenius Kabi, Bad Homburg, Germany), imipenem (250 mg/L; Fresenius Kabi) and metronidazole (1 g/L; B. Braun, Melsungen, Germany) to drinking water (*ad libitum*). Microbiota-depleted mice were kept and handled under strict aseptic conditions. Three days before *C. jejuni* infection, the animals received autoclaved tap water.

### 2.3. Campylobacter jejuni Infection and Clove EO Treatment

*C. jejuni* strain 81-176 was thawed from frozen stocks and grown on Columbia agar (with 5% sheep blood) and selective Karmali agar plates (both from Oxoid, Wesel, Germany). Age- and sex-matched microbiota-depleted IL-10^−/−^ mice (4-month-old littermates) were infected perorally with 10^9^ colony forming units (CFU) of the pathogen on days 0 and 1 by gavage. For one experiment, mice were assigned to three groups: uninfected und untreated (i.e., naive) group (n = 4); *C. jejuni* infected group treated with placebo (n = 4); and the infected group treated with clove EO (100 mg per kg body weight per day, n = 4). Treatment with clove EO (purchased from Sigma-Aldrich, Munich, Germany) was performed from day 2 post-infection (p.i.) until the end of the observation period and applied to autoclaved tap water (final concentration of 5 g/L; *ad libitum*). The placebo control mice received autoclaved tap water only.

### 2.4. Gastrointestinal C. jejuni Loads

After *C. jejuni* infection, the pathogen loads were determined in fecal samples daily, and upon necropsy in luminal samples from the stomach, duodenum, ileum, and colon by culture as described previously [39,41]. In brief, intraluminal gastrointestinal samples were homogenized in sterile phosphate-buffered saline (PBS, Thermo Fisher Scientific, Waltham, MA, USA) with a sterile pistil and serial dilutions plated onto Karmali agar (Oxoid, Wesel, Germany) and incubated under microaerophilic conditions for at least 48 h (37 °C). The detection limit of viable pathogens was 100 CFU per g.

### 2.5. Clinical Conditions

Immediately before and after infection, we quantitatively surveyed the daily clinical outcome of mice in a blinded fashion by using a cumulative clinical score (maximum 12 points), addressing the abundance of blood in feces (0: no blood; 2: microscopic detection of blood by the Guajac method using Hemoccult, Beckman Coulter/PCD, Germany; 4: macroscopic blood visible), the stool consistency (0: formed feces; 2: pasty feces; 4: liquid feces) and the clinical aspect (i.e., wasting symptoms; 0: normal; 1: ruffled fur; 2: less locomotion; 3: isolation; 4: severely compromised locomotion, pre-final aspect) as described earlier 2014 [42].

### 2.6. Sampling Procedures

On day 6 p.i., mice were sacrificed by CO_2_ asphyxiation. Cardiac blood (for serum cytokine measurements), ex vivo biopsies from liver, kidneys, spleen, ileum, and colon as well as luminal samples from stomach, duodenum, ileum, and colon were derived under aseptic conditions. From each mouse, colonic samples were collected in parallel for subsequent microbiological and immunohistopathological analyses.

### 2.7. Histopathology

Histopathological analyses were performed in colonic ex vivo biopsies that had been immediately fixed in 5% formalin and embedded in paraffin. Sections (5 µm) were stained with hematoxylin and eosin (H&E), examined by light microscopy (100× magnification), and histopathological changes in the large intestines quantitatively assessed with histopathological scores [43]: Score 1, minimal inflammatory cell infiltrates in the mucosa with intact epithelium. Score 2, mild inflammatory cell infiltrates in the mucosa and submucosa with mild hyperplasia and mild goblet cell loss. Score 3, moderate inflammatory cell infiltrates in the mucosa with moderate goblet cell loss. Score 4, marked inflammatory cell infiltration in the mucosa and submucosa with marked goblet cell loss, multiple crypt abscesses, and crypt loss.

### 2.8. In Situ Immunohistochemistry

Quantitative in situ immunohistochemical analyses were performed in colonic ex vivo biopsies following immediate fixation in 5% formalin and embedding in paraffin as recently reported [44,45]. In brief, to detect apoptotic epithelial cells, macrophages/monocytes, T lymphocytes, regulatory T cells, and B lymphocytes, colonic paraffin sections (5 µm) were stained with primary antibodies against cleaved caspase-3 (Asp175, Cell Signaling, Beverly, MA, USA, 1:200), F4/80 (no. 14-4801, clone BM8, eBioscience, San Diego, CA, USA, 1:50), CD3 (no. N1580, Dako, 1:10), FOXP3 (clone FJK-165, no. 14-5773, eBioscience, 1:100), and B220 (no. 14-0452-81, eBioscience; 1:200), respectively. Positively stained cells were quantitated by a blinded independent investigator applying light microscopy. The average number of respective positively stained cells in each sample was determined within at least six high power fields (HPF, 0.287 mm^2^, 400× magnification).

### 2.9. Pro-Inflammatory Mediators

Intestinal ex vivo biopsies were collected from the colon and ileum (longitudinally cut strips of approximately 1 cm^2^, washed in PBS) and extra-intestinal samples taken from the liver (approximately 1 cm^3^), the kidney (one half after the longitudinal cut) and the spleen (one third) subsequently transferred to 24-flat-bottom well culture plates (Thermo Fisher Scientific, Waltham, MA, USA) containing 500 mL serum-free RPMI 1640 medium (Thermo Fisher Scientific, Waltham, MA, USA) supplemented with penicillin (100 µg/mL) and streptomycin (100 µg/mL; Biochrom, Berlin, Germany). After an 18-h incubation period at 37 °C, respective culture supernatants and serum samples were tested for interferon-γ (IFN-γ), tumor necrosis factor-α (TNF-α), monocyte chemoattractant protein-1 (MCP-1), and interleukin-6 (IL-6) by the Mouse Inflammation Cytometric Bead Assay (CBA; BD Biosciences, Germany) in a BD FACSCanto II flow cytometer (BD Biosciences). 

### 2.10. Statistical Analyses

Medians and significance levels were calculated using GraphPad Prism (version 8; San Diego, CA, USA). Normalization of data was surveyed by the Anderson-Darling test. The Mann–Whitney test was applied for pairwise comparisons of not normally distributed data. For multiple comparisons, the one-way ANOVA with Tukey post-correction (for normally distributed data) and the Kruskal-Wallis test with Dunn’s post-correction (for not normally distributed data) were performed. Two-sided probability (*p*) values ≤ 0.05 were considered significant. Definite outliers were removed after being identified by the Grubb’s test (α = 0.001). Data were pooled from four independent experiments.

## 3. Results

### 3.1. Gastrointestinal Pathogen Burdens Following Clove EO Treatment of C. jejuni Infected IL-10^−/−^ Mice 

**Figure 1:**
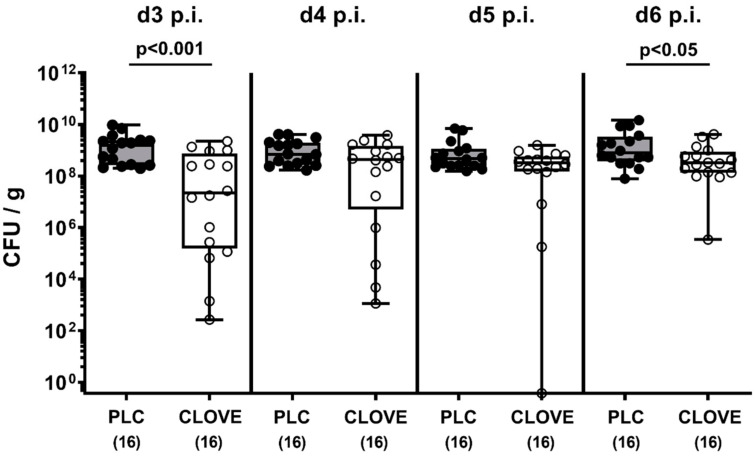
Kinetic survey of fecal pathogen burdens following clove essential oil (EO) treatment of *Campylobacter* (*C.*) *jejuni* infected interleukin-10 deficient (IL-10^−/−^) mice. Microbiota-depleted IL-10^−/−^ mice were perorally infected with *C. jejuni* strain 81-176 on day (d) 0 and d1. From d2 until d6 post-infection (p.i.), mice were perorally challenged with clove EO or received placebo (PLC) via drinking water. The pathogen numbers were determined in fecal samples over time by culture and expressed as colony forming units (CFU) per g). Box plots indicate the 75th and the 25th percentiles of the median (black bar within box). The total range, significance levels (*p* values) determined by the Mann–Whitney U test and the total numbers of analyzed mice (in parentheses) are given. Data were pooled from four independent experiments.

Microbiota-depleted IL-10^−/−^ mice were perorally infected with *C. jejuni* strain 81-176 and subjected to either clove EO or placebo treatment via drinking water starting on day 2 p.i. The cultural survey of the fecal pathogen loads revealed that on day 3 and day 6 p.i. clove EO-treated mice harbored approximately 2.0 and 0.5 log orders of magnitude lower median *C. jejuni* cell numbers in their intestines, respectively, as compared to placebo control mice (*p* < 0.001 and *p* < 0.05, respectively; Figure 1). 

**Figure 2:**
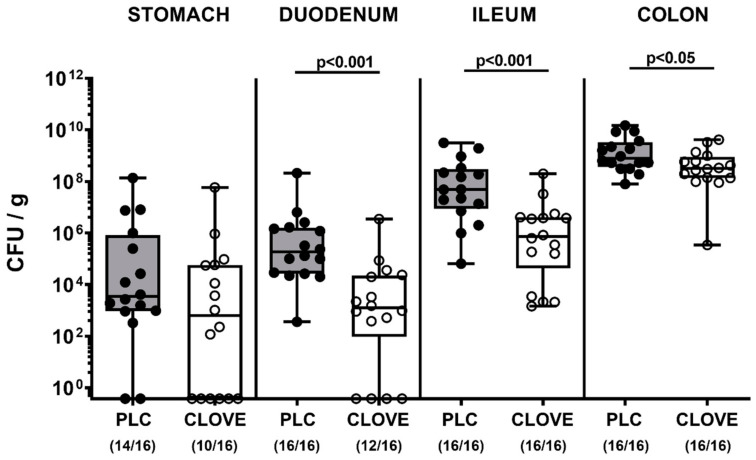
Gastrointestinal pathogen burdens following clove EO treatment of *C. jejuni* infected IL-10^−/−^ mice. Microbiota-depleted IL-10^−/−^ mice were perorally infected with *C. jejuni* strain 81-176 on day (d) 0 and d1. From d2 until d6 post-infection, mice were perorally challenged with clove EO or received placebo (PLC) via drinking water. On d6 post-infection, the pathogen numbers were determined in luminal samples taken from distinct gastrointestinal parts by culture and expressed as colony forming units per g, CFU/g). Box plots indicate the 75th and the 25th percentiles of the median (black bar within box). The total range, significance levels (*p* values) determined by the Mann–Whitney U test and the number of pathogen-positive out of the total number of analyzed samples (in parentheses) are given. Data were pooled from four independent experiments.

On the day of necropsy (i.e., day 6 p.i.), we further surveyed the pathogen burdens within the gastrointestinal tract. Although in the stomach lumen the *C. jejuni* loads were comparable in both cohorts, clove EO-treated mice harbored slightly lower pathogen numbers in their colonic lumen as compared to placebo control animals (*p* < 0.05; Figure 2). In both the proximal and distal small intestines these differences were, however, even more pronounced as indicated by more than two log orders of magnitude lower *C. jejuni* cell counts in the duodenum and the terminal ileum of clove EO versus placebo treated mice on day 6 p.i. (*p* < 0.001; Figure 2). Hence, clove EO treatment resulted in decreased pathogen loads in the small and large intestines of *C. jejuni* infected IL-10^−/−^ mice. 

### 3.2. C. jejuni Induced Clinical Conditions Following Clove EO Treatment of Infected IL-10^−/−^ Mice

**Figure 3:**
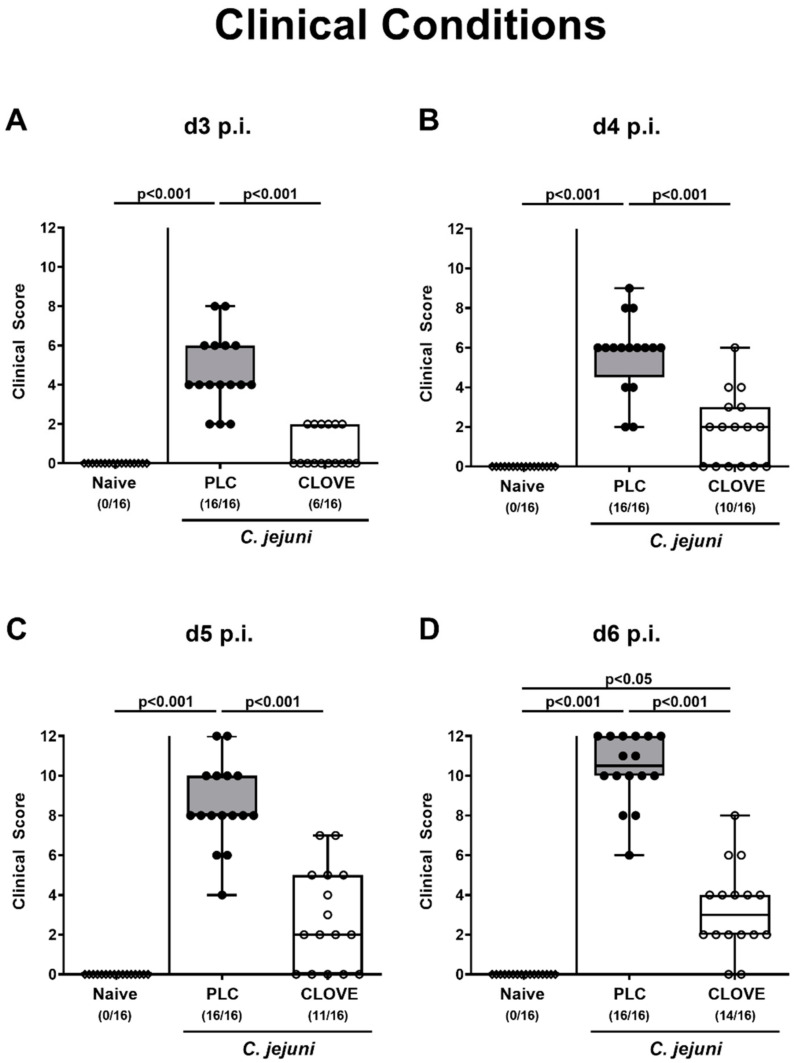
Kinetic survey of *C. jejuni* induced clinical conditions following clove EO treatment of infected IL-10^−/−^ mice. Microbiota-depleted IL-10^−/−^ mice were perorally infected with *C. jejuni* strain 81-176 on day (d) 0 and d1. From d2 until d6 post-infection (p.i.), mice were perorally challenged with clove EO or received placebo (PLC) via drinking water. The clinical conditions of mice were quantitatively determined over time, i.e., on (**A**) d3, (**B**) d4, (**C**) d5 and (**D**) d6 p.i. by using a clinical scoring system by using a clinical scoring system. Box plots indicate the 75th and the 25th percentiles of the median (black bar within box). Naive mice were included as negative control animals. The total range, significance levels (*p* values) determined by the Kruskal-Wallis test and Dunn’s post-correction and the number of mice with clinical signs out of the total number of analyzed animals (in parentheses) are given. Data were pooled from four independent experiments.

We further quantitatively surveyed the pathogen-induced disease in clove EO-treated infected mice over time. As early as 24 h after initiating the clove EO treatment (i.e., day 3 p.i.), mice displayed less severe clinical signs of campylobacteriosis such as diarrhea and fecal blood as compared to placebo counterparts (*p* < 0.01–0.001; Figure 3, Figure S1A–H) that did not differ from naive animals until day 5 p.i. (not significant (n.s.); Figure 3, Figure S1A–H). At the end of the observation period on day 6 p.i., when all placebo control mice were suffering from full-blown disease including wasting and bloody diarrhea, clove EO-treated mice were far less severely compromised (*p* < 0.001; Figure 3, Figure S1A–M) and remarkably, 12.5% of mice did not display any clinical signs at all (Figure 3). Hence, clove EO treatment resulted in a better clinical outcome of *C. jejuni* infected IL-10^−/−^ mice.

### 3.3. C. jejuni Induced Microscopic Inflammatory Sequelae Following Clove EO Treatment of Infected IL-10^−/−^ Mice

**Figure 4:**
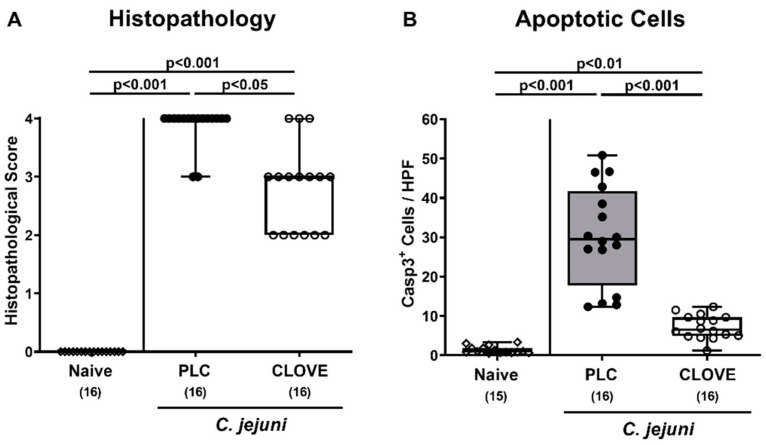
*C. jejuni* induced microscopic inflammatory sequelae following clove EO treatment of infected IL-10^−/−^ mice. Microbiota-depleted IL-10^−/−^ mice were perorally infected with *C. jejuni* strain 81-176 on day (d) 0 and d1. From d2 until d6 post-infection (p.i.), mice were perorally challenged with clove EO or received placebo (PLC) via drinking water. On day 6 p.i., (**A**) colonic histopathological changes were quantified in hematoxylin and eosin-stained colonic paraffin sections by using histopathological scores. Furthermore, (**B**) the average numbers of apoptotic colonic epithelial cells were assessed microscopically from six high power fields (HPF, 400 × magnification) per animal in paraffin sections positive for cleaved caspase3 (Casp3^+^). Box plots indicate the 75th and the 25th percentiles of the median (black bar within box). Naive mice were included as negative control animals. The total range, significance levels (*p* values) determined by the ANOVA test with Tukey post-correction or by the Kruskal-Wallis test and Dunn’s post-correction and the total numbers of analyzed mice (in parentheses) are given. Definite outliers were removed after being identified by the Grubb’s test (α = 0.001). Data were pooled from four independent experiments.

We then addressed whether clove EO treatment could improve microscopic inflammatory sequelae of *C. jejuni* infection. Therefore, histopathological changes were quantified in the large intestines using histopathological scores. Mice from the placebo cohort displayed severe histopathological inflammatory responses upon *C. jejuni* infection, whereas clove EO-treated mice exhibited lower histopathological scores on day 6 p.i., indicative for moderate colonic inflammation (*p* < 0.05; Figure 4A). In support, numbers of apoptotic colonic epithelial cells had increased within 6 days following *C. jejuni* infection of mice from both cohorts (*p* < 0.01–0.001), but less distinctly in clove EO versus placebo treated mice (*p* < 0.001; Figure 4B). Of note, median numbers of cleaved caspase3^+^ colonic epithelial cells were approximately four times higher in placebo versus clove EO-treated mice (Figure 4B). Hence, clove EO treatment resulted in less severe microscopic inflammatory sequelae in *C. jejuni* infected IL-10^−/−^ mice. 

### 3.4. C. jejuni Induced Immune Cell Responses Following Clove EO Treatment of Infected IL-10^−/−^ Mice

**Figure 5:**
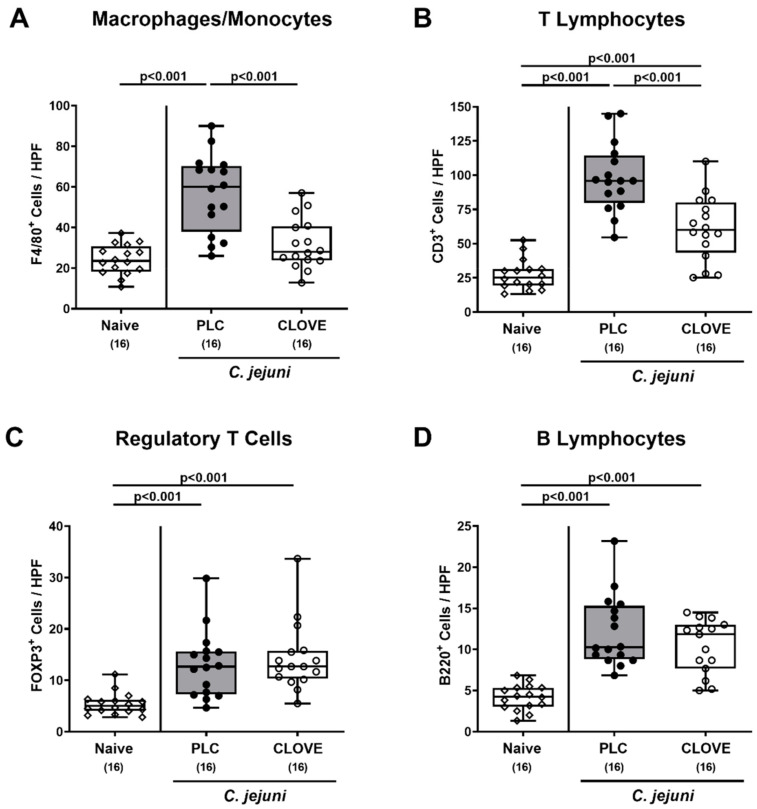
*C. jejuni* induced immune cell responses following clove EO treatment of infected IL-10^−/−^ mice. Microbiota-depleted IL-10^−/−^ mice were perorally infected with *C. jejuni* strain 81-176 on day (d) 0 and d1. From d2 until d6 post-infection (p.i.), mice were perorally challenged with clove EO or received placebo (PLC) via drinking water. On day 6 p.i., the average numbers of (**A**) macrophages and monocytes (F4/80^+^), (**B**) T lymphocytes (CD3^+^), (**C**) regulatory T cells (FOXP3^+^) and (**D**) B lymphocytes (B220^+^) per animal were determined in immunohistochemically stained colonic paraffin sections from six high power fields (HPF, 400× magnification). Box plots indicate the 75th and the 25th percentiles of the median (black bar within box). Naive mice were included as negative control animals. The total range, significance levels (*p* values) determined by the Kruskal-Wallis test and Dunn’s post-correction and the total numbers of analyzed mice (in parentheses) are given. Data were pooled from four independent experiments.

To address potential immune-modulatory properties of clove EO treatment during acute campylobacteriosis, we performed in situ immunohistochemical staining of colonic paraffin sections with antibodies against distinct immune cell subsets. On day 6 p.i., higher numbers of F4/80^+^ macrophages and monocytes could be enumerated in the colonic mucosa and lamina propria of placebo, but not of clove EO-treated mice when compared to naive control animals (*p* < 0.001 versus placebo; Figure 5A). Furthermore, *C. jejuni* infection was accompanied by increases in colonic CD3^+^ T cell numbers that were, however, less pronounced in mice from the clove EO cohort as compared to placebo counterparts (*p* < 0.001; Figure 5B). When assessing FOXP3^+^ regulatory T cells and B220^+^ B lymphocytes, comparably elevated cell numbers could be observed on day 6 p.i., irrespective of the treatment regimen (*p* < 0.001 versus naive; Figure 5C,D). Hence, clove EO treatment dampened *C. jejuni* induced increases in colonic macrophages/monocytes and T lymphocytes.

### 3.5. C. jejuni Induced Intestinal Proinflammatory Cytokine Secretion Following Clove EO Treatment of Infected IL-10^−/−^ Mice 

**Figure 6:**
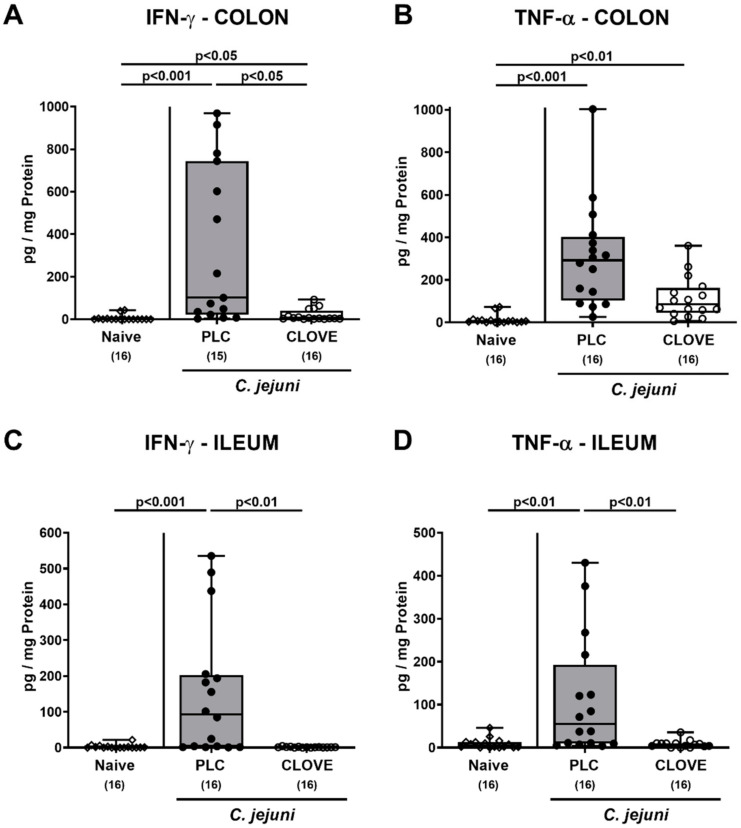
*C. jejuni* induced intestinal proinflammatory cytokine secretion following clove EO treatment of infected IL-10^−/−^ mice. Microbiota-depleted IL-10^−/−^ mice were perorally infected with *C. jejuni* strain 81-176 on day (d) 0 and d1. From d2 until d6 post-infection (p.i.), mice were perorally challenged with clove EO or received placebo (PLC) via drinking water. On day 6 p.i., (**A**,**C**) IFN-γ and (**B**,**D**) TNF-α concentrations were measured were measured in ex vivo biopsies derived from the (**A**,**B**) colon and (**C**,**D**) ileum. Box plots indicate the 75th and the 25th percentiles of the median (black bar within box). Naive mice were included as negative control animals. The total range, significance levels (*p* values) determined by the Kruskal-Wallis test and Dunn’s post-correction and the total numbers of analyzed mice (in parentheses) are given. Data were pooled from four independent experiments.

We next addressed the impact of clove EO treatment on *C. jejuni* induced proinflammatory cytokine secretion in the intestinal tract. On day 6 p.i., increased IFN-γ and TNF-α concentrations could be measured in colonic ex vivo biopsies (*p* < 0.05–0.001; Figure 6A,B). In clove EO-treated mice, however, colonic IFN-γ concentrations were lower as compared to placebo controls (*p* < 0.05; Figure 6A), whereas a trend towards lower TNF-α levels could be observed in the former versus the latter (n.s. due to high standard deviations; Figure 6B). In the ileum of placebo as opposed to clove EO-treated mice, elevated IFN-γ and TNF-α concentration were measured on day 6 p.i. (*p* < 0.01–0.001; Figure 6C,D). Hence, clove EO treatment of *C. jejuni* infected mice was associated with decreased intestinal proinflammatory cytokine secretion. 

### 3.6. C. jejuni Induced Extra-Intestinal Proinflammatory Cytokine Secretion Following Clove EO Treatment of Infected IL-10^−/−^ Mice 

**Figure 7:**
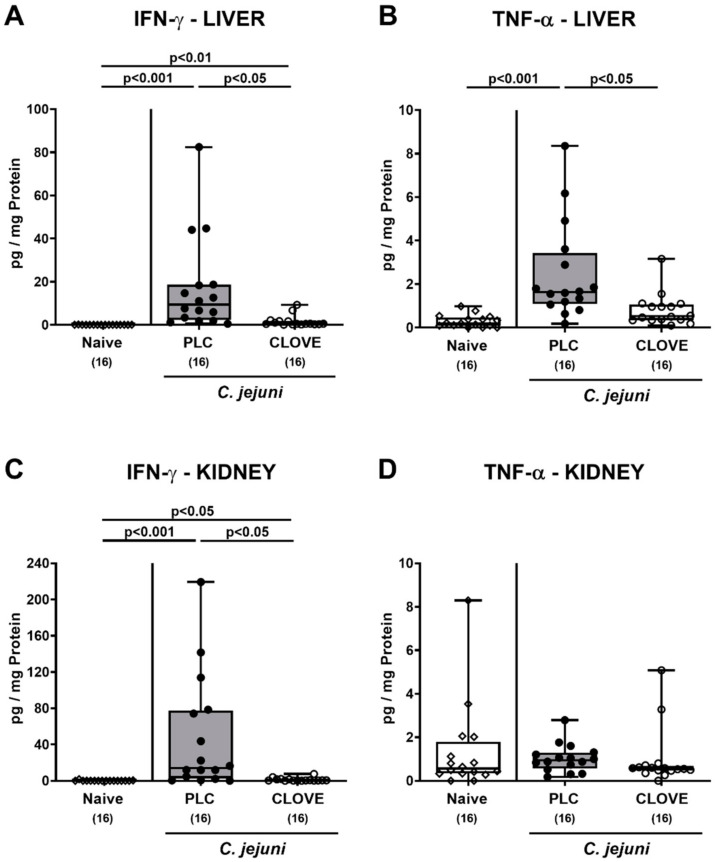
*C. jejuni* induced extra-intestinal proinflammatory cytokine secretion following clove EO treatment of infected IL-10^−/−^ mice. Microbiota-depleted IL-10^−/−^ mice were perorally infected with *C. jejuni* strain 81-176 on day (d) 0 and d1. From d2 until d6 post-infection (p.i.), mice were perorally challenged with clove EO or received placebo (PLC) via drinking water. On day 6 p.i., (**A**,**C**) IFN-γ and (**B**,**D**) TNF-α concentrations were measured in ex vivo biopsies derived from the (**A**,**B**) liver and (**C**,**D**) kidneys. Box plots indicate the 75th and the 25th percentiles of the median (black bar within box). Naive mice were included as negative control animals. The total range, significance levels (*p* values) determined by the Kruskal-Wallis test and Dunn’s post-correction and the total numbers of analyzed mice (in parentheses) are given. Data were pooled from four independent experiments.

We next addressed whether the immune-modulatory effects of clove EO were effective beyond the intestinal tract. Therefore, proinflammatory cytokines were measured in extra-intestinal ex vivo biopsies. On day 6 p.i., pathogen-induced increases in hepatic and renal IFN-γ were measured in placebo and clove EO-treated mice (*p* < 0.05–0.001), but to a lesser extent in the latter versus the former (*p* < 0.05; Figure 7A,C). Moreover, *C. jejuni* infection resulted in enhanced TNF-α secretion in livers taken from mice of the placebo, but not the clove EO cohort (n.s. versus naive; *p* < 0.05 versus placebo; Figure 7B). In the kidneys, however, TNF-α concentrations were comparable in all groups (Figure 7D). 

**Figure 8:**
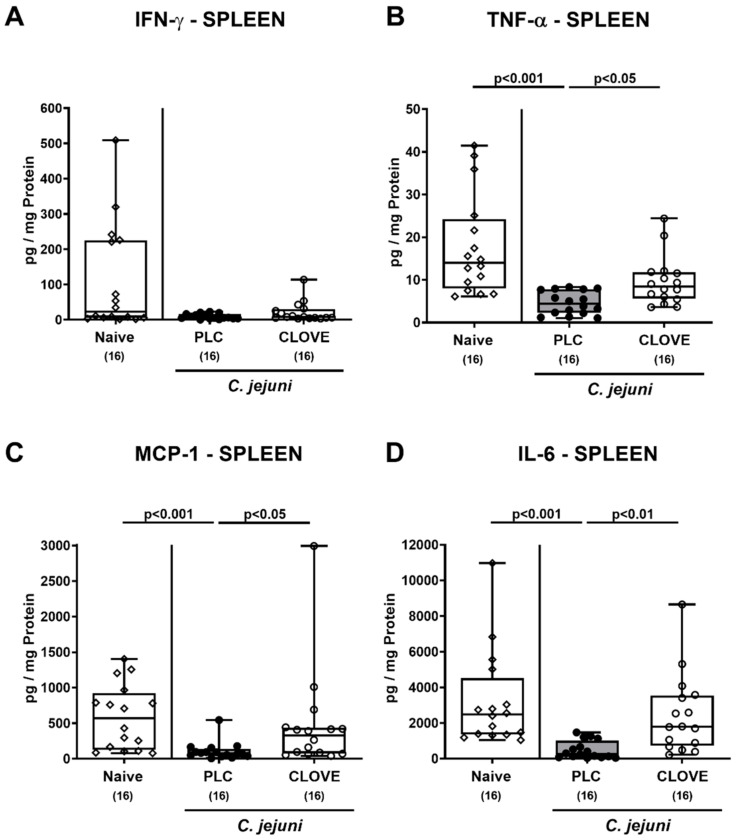
*C. jejuni* induced splenic proinflammatory mediator secretion following clove EO treatment of infected IL-10^−/−^ mice. Microbiota-depleted IL-10^−/−^ mice were perorally infected with *C. jejuni* strain 81-176 on day (d) 0 and d1. From d2 until d6 post-infection (p.i.), mice were perorally challenged with clove EO or received placebo (PLC) via drinking water. On day 6 p.i., (**A**) IFN-γ, (**B**) TNF-α, (**C**) MCP-1 and (**D**) IL-6 concentrations were measured in ex vivo biopsies derived from the spleen. Box plots indicate the 75th and the 25th percentiles of the median (black bar within box). Naive mice were included as negative control animals. The total range, significance levels (*p* values) determined by the Kruskal-Wallis test and Dunn’s post-correction and the total numbers of analyzed mice (in parentheses) are given. Data were pooled from four independent experiments.

We further addressed proinflammatory mediator secretion in spleens and found comparable IFN-γ concentrations (n.s.; Figure 8A). In placebo, but not clove EO-treated mice, however, lower splenic TNF-α, MCP-1, and IL-6 concentrations could be determined on day 6 p.i. as compared to naive controls (*p* < 0.001 versus naive; *p* < 0.05–0.01 versus clove EO; Figure 8B–D). 

**Figure 9:**
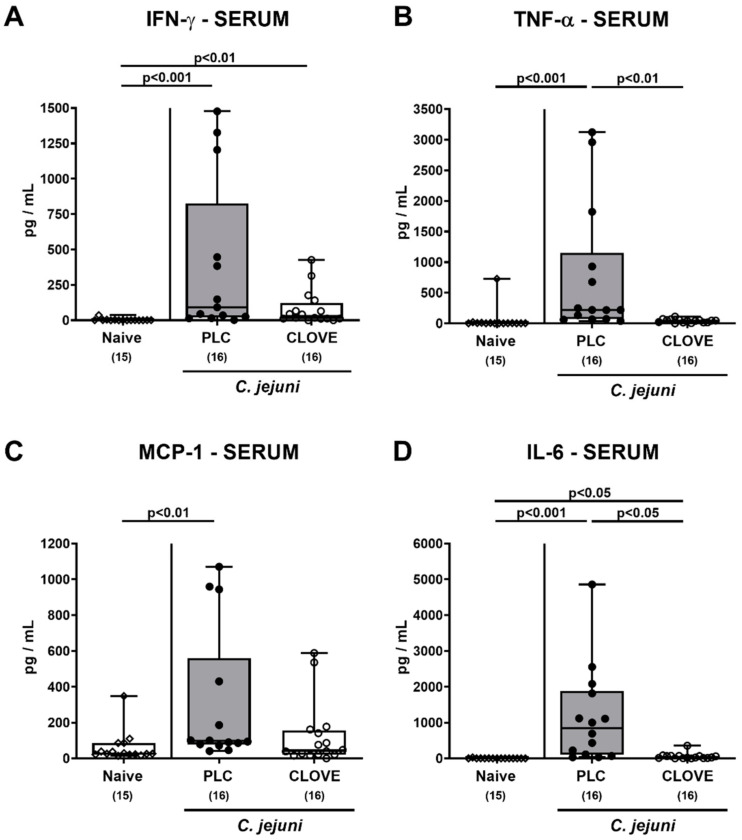
*C. jejuni* induced systemic proinflammatory mediator secretion following clove EO treatment of infected IL-10^−/−^ mice. Microbiota-depleted IL-10^−/−^ mice were perorally infected with *C. jejuni* strain 81-176 on day (d) 0 and d1. From d2 until d6 post-infection (p.i.), mice were perorally challenged with clove EO or received placebo (PLC) via drinking water. On day 6 p.i., (**A**) IFN-γ, (**B**) TNF-α, (**C**) MCP-1 and (**D**) IL-6 concentrations were measured in serum samples. Box plots indicate the 75th and the 25th percentiles of the median (black bar within box). Naive mice were included as negative control animals. The total range, significance levels (*p* values) determined by the Kruskal-Wallis test and Dunn’s post-correction and the total numbers of analyzed mice (in parentheses) are given. Definite outliers were removed after being identified by the Grubb’s test (α = 0.001). Data were pooled from four independent experiments.

When assessing systemic proinflammatory mediator secretion, increased IFN-γ concentrations were measured in serum samples taken from both placebo and clove EO-treated mice on day 6 p.i., but with a trend towards lower concentrations in the latter as compared to the former (n.s. due to high standard deviations; Figure 9A). Furthermore, *C. jejuni* infection was associated with elevated TNF-α and MCP-1 serum concentrations in placebo control mice only (*p* < 0.001 and *p* < 0.01, respectively; Figure 9B,C), whereas pathogen-induced IL-6 serum secretion was more enhanced in placebo as compared to clove EO-treated mice (*p* < 0.05; Figure 9D). Hence, clove EO exerted potent immune-modulatory effects in extra-intestinal and remarkably, even systemic compartments of *C. jejuni* infected IL-10^−/−^ mice.

## 4. Discussion

Our first preclinical evaluation of clove EO as a promising treatment option for acute campylobacteriosis revealed not only intestinal, but also extra-intestinal and remarkably, even systemic anti-inflammatory effects. The decreased pathogen loads in both the small and large intestines and lowered proinflammatory immune responses in clove EO versus placebo treated *C. jejuni* infected IL-10^−/−^ mice are indicative for combined antimicrobial and immune-modulatory effects of the natural compound. Although the underlying mechanisms were not in the focus of our investigations, direct antipathogenic effects of clove EO are supported by the earlier findings that clove EO is able to kill *C. jejuni* and other bacteria by the concerted action of membrane destruction and intracellular ROS formation. By the application of 5 g/L clove EO in drinking water we assured that the drug concentrations taken up by infected mice were above the minimum inhibitory concentration (MIC) values which are required to kill *C. jejuni* in vitro [29]. This was further confirmed by the MIC of eugenol constituting the major compound of clove EO against the *C. jejuni* strain 81-176 (i.e., 0.1 g/L; data not shown). Thus, the here applied clove EO concentrations were sufficient to induce the antimicrobial effects of the natural compound against *C. jejuni* including the potent inhibition of pathogenic motility, the deformation of the spiral-shaped morphology and the reduction of virulence [31,32,33]. Bacterial factors inhibited by eugenol include adhesins, invasins and quorum sensing regulators which are required for bacterial colonization of the intestinal tract and for the onset of infection. Hence, clove EO treatment exerts multi-facetted effects. Beyond direct killing of a part of the *C. jejuni* population it reduces the virulence of surviving pathogens. We therefore hypothesize that despite the relatively high numbers of viable *C. jejuni* bacteria we isolated from the colonic lumen of cove EO-treated mice at day 6 p.i. (i.e., approximately 10^8^ CFU/g), the bacteria were not able to induce acute campylobacteriosis as opposed to the placebo cohort. In support, our recent investigations revealed that non-motile mutants of *C. jejuni* are apathogenic in microbiota-depleted IL-10^−/−^ mice despite high intestinal colonization densities of the bacteria [20].

In view of the preclinical impact of our investigations, it is important to note that clove EO treatment resulted not only in reduced intestinal pathogen loads but also in a better clinical outcome upon *C. jejuni* infection. Remarkably, a prominent alleviation of *C. jejuni* induced clinical signs such as fecal blood and diarrhea could be observed as early as 24 h after initiation of the clove EO treatment. During the later course of campylobacteriosis (i.e., at day 6 p.i.), also wasting symptoms indicative for acute systemic disease were far less pronounced in clove EO as compared to placebo mice. In support, the disease-alleviating effects of the natural compound could also be observed on microscopic level given less pronounced histopathological changes in the large-intestinal mucosa and lamina propria and markedly lower apoptotic colonic epithelial cell counts in clove EO versus placebo treated mice, both of which constituting hallmarks of intestinal campylobacteriosis [39]. In support, anti-inflammatory effects of clove EO, of eugenol and of other eugenol containing plant extracts have been demonstrated during experimental intestinal inflammation such as chemically induced colitis in rats [37,38,46]. It is noteworthy that the treatment of murine ulcerative colitis with eugenol containing plant extracts resulted in anti-inflammatory effects that were comparable to those obtained by prednisolone treatment [46]. Furthermore, the potent anti-apoptotic effects of eugenol in the intestinal tract were independently confirmed in a rat model of intestinal ischemia and reperfusion injury [47].

The immune-modulatory properties of clove EO treatment included the dampening of *C. jejuni* induced intestinal immune cell responses as indicated by decreased colonic numbers of innate and adaptive immune cell subsets such as macrophages/monocytes and T lymphocytes, respectively, whereas regulatory T cell and B lymphocyte counts were unaffected. In support of these findings, clove EO treatment of *C. jejuni* infected mice was associated with decreased intestinal secretion of the proinflammatory key cytokines IFN-γ and TNF-α. The potent immune-modulatory properties of clove EO were not restricted to the intestinal tract, however, as indicated by less pronounced proinflammatory cytokine secretion in livers and kidneys taken from the verum versus placebo treated mice. Strikingly, clove EO treatment exerted also immune-modulatory effects at systemic tissue sites. Upon *C. jejuni* infection TNF-α, IL-6 and MCP-1 concentrations were lower in spleens taken from placebo as opposed to clove EO-treated mice when compared to uninfected control animals, indicative for enhanced recruitment of splenic leukocytes to infected compartments of the former versus the latter. Furthermore, in support of the less distinct wasting symptoms observed at day 6 following infection of mice from the clove EO versus the placebo cohort, lower TNF-α, IL-6, and MCP-1 concentrations were measured in serum samples taken from the former versus the latter.

Although the mechanisms underlying the immune-modulatory effects of clove EO have not been investigated in more detail in our present preclinical intervention study, it is tempting to speculate that the utmost potent antioxidant properties of eugenol might contribute to the ameliorated apoptotic and proinflammatory responses during acute murine campylobacteriosis (reviewed by Fujisawa and Murakami, 2016 [36]). The multifaceted antioxidant activities of a single molecule exerted by dimerization, recycling, and chelation of ROS in concert with direct antimicrobial and anti-virulence properties render eugenol and clove EO ideal candidates for the treatment and prevention of campylobacteriosis in humans. With respect to the pivotal immunopathological role of LOS during *C. jejuni* infection it seems noteworthy that eugenol showed potent inhibition of LPS induced inflammatory responses in porcine intestinal cells [48] and in murine lung injury [49]. 

When considering the implementation of immune-modulatory agents in the treatment of infectious diseases, one needs to take into consideration, however, that the host immune responses upon infections display a dichotomous mode of action, namely combating the pathogen on one side but by the expenses of potentially harmful collateral damages to the host on the other. Our interventive strategy to counteract the massive intestinal inflammation following *Campylobacter*-induced innate immune responses by immune-modulatory natural agents leading to rather moderate immune suppression is based on the earlier finding by Sun et al., 2012. This study revealed that immune-suppression of mice by rapamycin applied before or after *C. jejuni* infection blocked both *C. jejuni* induced intestinal inflammation and bacterial accumulation [21]. Hence, the excessive innate immune responses constitute promising targets for novel intervention strategies in the combat of acute campylobacteriosis.

A potential application of the here presented preclinical data to human medicine (from bench and mouse facility to bedside) highly depends on the pharmacological and toxicological safety evaluation of clove EO in rodent disease models. In this context, results from a corresponding study revealed that peroral treatment with Clovinol, a powder derived from a defined clove bud extract, resulted in significant antioxidant and anti-inflammatory effects in carrageenan-induced paw swelling and ethanol-driven stomach ulcerations in mice and rats, respectively [50]. Investigations of Clovinol toxicity showed that the drug can be considered to be safe and suitable for further clinical investigations at dosages applied here for clove EO treatment of acute murine campylobacteriosis (i.e., 100 mg/kg body weight/day).

## 5. Conclusions

Our preclinical intervention study indicates for the first time that clove EO has a promising therapeutic potential for the treatment and, presumably, prophylaxis of acute campylobacteriosis and might prevent from post-infectious morbidities. Given the safety profile and the long-term use of this compound in traditional medicine the preclinical data obtained here might pave the way to future clinical studies analyzing the efficacy of clove EO treatment against human campylobacteriosis.

## Data Availability

The data presented in this study are available on request from the corresponding author.

## Supplementary Materials

The following are available online at https://www.mdpi.com/article/10.3390/microorganisms9040735/s1, Supplementary Figure S1: Kinetic survey of *C. jejuni* induced clinical signs following clove EO treatment of infected IL-10^−/−^ mice.
