# Supplementary material for: Peroral Clove Essential Oil Treatment Ameliorates Acute Campylobacteriosis—Results from a Preclinical Murine Intervention Study
Source: Microorganisms. 2021 Mar 31;9(4):735. doi: 10.3390/microorganisms9040735 (PMC8066448; doi:10.3390/microorganisms9040735)
Supplement: Supplementary file 1 [file microorganisms-09-00735-s001.pdf]

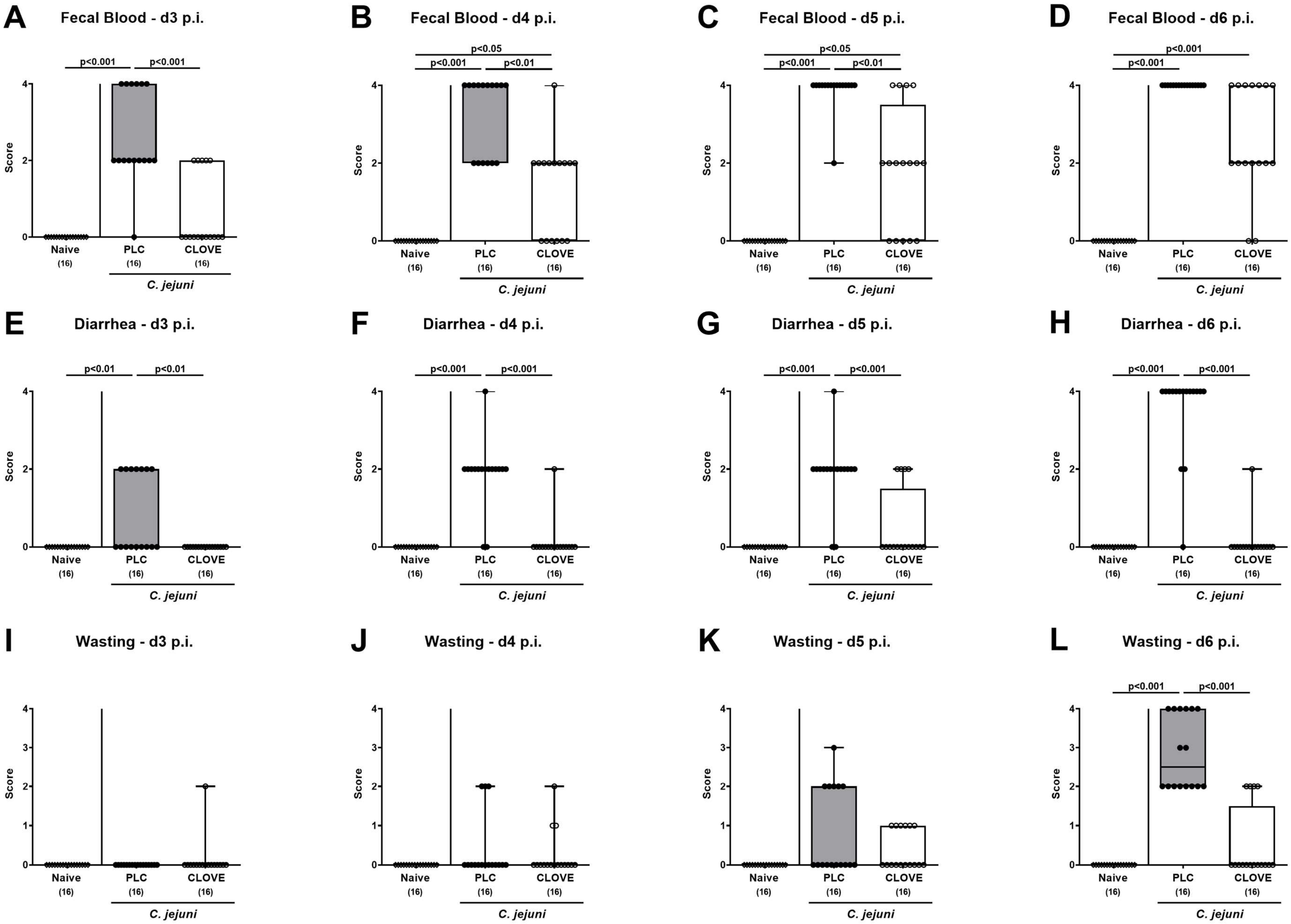

A
Fecal Blood - d3 p.i.
p<0.001
p<0.001
B
Fecal Blood - d4 p.i.
p<0.05
p<0.001
p<0.01
C
Fecal Blood - d5 p.i.
p<0.05
p<0.001
p<0.01
D
Fecal Blood - d6 p.i.
p<0.001
p<0.001
E
Diarrhea - d3 p.i.
p<0.01
p<0.01
F
Diarrhea - d4 p.i.
p<0.001
p<0.001
G
Diarrhea - d5 p.i.
p<0.001
p<0.001
H
Diarrhea - d6 p.i.
p<0.001
p<0.001
I
Wasting - d3 p.i.
J
Wasting - d4 p.i.
K
Wasting - d5 p.i.
L
Wasting - d6 p.i.
p<0.001
p<0.001
Score
Naive (16)
PLC (16)
CLOVE (16)
C. jejuni

Supplementary Figure S1. Kinetic survey of *C. jejuni* induced clinical signs following clove EO treatment of infected IL-10$^{-/-}$ mice. Microbiota-depleted IL-10$^{-/-}$ mice were perorally infected with *C. jejuni* strain 81-176 on day (d) 0 and d1. From d2 until d6 post-infection (p.i.), mice were perorally challenged with clove EO or received placebo (PLC) via the drinking water. Pathogen-induced clinical signs such as **(A–D)** the abundance of fecal blood, **(E–H)** diarrhea and **(I–L)** wasting symptoms were quantitatively determined over time by using a clinical scoring system. Naive mice were included as negative control animals. Box plots indicate the 75th and the 25th percentiles of the median (black bars within box). The total range, significance levels (p values) determined by the Kruskal-Wallis test and Dunn's post-correction and the total numbers of analyzed mice (in parentheses) are given. Data were pooled from four independent experiments.
